# Supercritical CO_2_ Extraction of *Narcissus poeticus* L. Flowers for the Isolation of Volatile Fragrance Compounds

**DOI:** 10.3390/molecules27020353

**Published:** 2022-01-06

**Authors:** Renata Baranauskienė, Petras Rimantas Venskutonis

**Affiliations:** Department of Food Science and Technology, Kaunas University of Technology, LT-50254 Kaunas, Lithuania; renata.baranauskiene@ktu.lt

**Keywords:** *Narcissus poeticus*, SFE-CO_2_ extraction, volatile composition, aroma compounds

## Abstract

The flowers of *Narcissus poeticus* are used for the isolation of valuable fragrance substances. So far, as the majority of these substances consist of volatile and sensitive to heat compounds, there is a need of developing effective methods for their recovery. In this study, freeze-dried *N. poeticus* inflorescences were extracted with pure supercritical CO_2_ (SFE-CO_2_) and its mixture with 5% co-solvent ethanol (EtOH) at 40 °C. Extract yields varied from 1.63% (12 MPa) to 3.12% (48 MPa, 5% EtOH). In total, 116 volatile compounds were identified by GC-TOF/MS in the extracts, which were divided into 20 different groups. Benzyl benzoate (9.44–10.22%), benzyl linoleate (1.72–2.17%) and benzyl alcohol (0.18–1.00%) were the major volatiles among aromatic compounds. The amount of the recovered benzyl benzoate in *N. poeticus* SFE-CO_2_ extracts varied from 58.98 ± 2.61 (24 MPa) to 91.52 ± 1.36 (48 MPa) mg/kg plant dry weight (pdw). α-Terpineol dominated among oxygenated monoterpenes (1.08–3.42%); its yield was from 9.25 ± 0.63 (12 MPa) to 29.88 ± 1.25 (48 MPa/EtOH) mg/kg pdw. Limonene was the major monoterpene hydrocarbon; *(3E)*-hexenol and heneicosanol dominated among alcohols and phenols; dihydroactinidiolide and 4,8,12,16-tetramethyl heptadecan-4-olide were the most abundant lactones; heptanal, nonanal, *(2E,4E)*-decadienal and octadecanal were the most abundant aldehydes. The most important prenol lipids were triterpenoid squalene, from 0.86 ± 0.10 (24 MPa) to 7.73 ± 0.18 (48 MPa/EtOH) mg/kg pdw and D-α-tocopherol, from 1.20 ± 0.04 (12 MPa) to 15.39 ± 0.31 (48 MPa/EtOH) mg/kg pdw. Aliphatic hydrocarbons (waxes) constituted the main part (41.47 to 54.93%) in the extracts; while in case of a 5% EtOH the percentage of alkanes was the lowest. The fraction of waxes may be removed for the separation of higher value fragrance materials. In general, the results obtained are promising for a wider application of SFE-CO_2_ for the recovery of fragrance substances from *N. poeticus* flowers.

## 1. Introduction

Aromatic herbs and flowers are an important source of natural ingredients for the development of flavours and fragrances. *Narcissus* is a genus of perennial spring flowering plants of the Monocotyledon family Amaryllidaceae. Carl Linnaeus originally defined six species of *Narcissus* in 1753, while *N. poeticus* was the first one described in his book Species Plantarum [1]. Nowadays the genus is generally considered to consist of about 12 sections with approximately 80 species. Poetic, commonly also called daffodil (*N. poeticus*), supposed (*N. pseudonarcissus*), tripod (*N. triandrus*), and bouquet (*N. tazetta*) narcissus are the most widely grown species, while cyclamen (*N. cyclamineus*), chaste (*N. papyraceus*), and fragrant (*Narcissus* × *odorus*) are less popular. Three species, namely white (*N. poeticus*), bouquet (*N. tazetta*) and yellow/supposed (*N. pseudonarcissus*) are most popular in Lithuania as ornamental plants in flower gardens, which also grow in old parks and meadows.

*N. poeticus* is a wild, bulbous herbaceous plant, growing up to 20–40 cm height. The leaves are radical, green, narrow and long; it blooms in late spring (April–May) with an extremely fragrant white flower per stem [2]. *N. poeticus* is thought to originate from the Middle East or Eastern Mediterranean; now it is spread all over Europe, particularly France, Spain, southern Italy and north-western Greece [3]. It is also naturalized in New Zealand, British Columbia, Canada, United States, North Africa, and Asia.

The plants of the Amaryllidaceae family have also been used as herbal remedies since ancient times. The *Narcissus* genus is a rich source of potentially valuable pharmaceuticals, such as alkaloids galanthamine, lycorine, isoquinolines narciclasine, pancratistatin, *(E)*-dihydronarciclasine, and their corresponding 7-deoxyanalogues, narcipavline, norlycoramine; some of them might be promising in the treatment of Alzheimer’s and oncological diseases [4,5,6,7,8]. Health beneficial chlorogenic (755.93 ± 4.06 μg/g), *p*-coumaric, ferulic acids and flavonoids (hyperoside, isoquercitrin, quercitrin) were reported in *N. poeticus* ethanolic extract from Romania [9].

Essential oils and extracts used in the perfumery industry are mostly produced from *N. jonquilla*, *N. poeticus*, *N. tazetta* and some other species. *N. poeticus* flowers as exceptionally fragrant material are cultivated in the Netherlands and southern France mainly for distilling essential oil (EO). Groom [10] described the aroma of the narcissus oil as resembling the combination of jasmine and hyacinth; therefore, its floral concrete or absolute has been used as a principal ingredient in ~11% of modern quality perfumes, including such famous names as “Fatale” or “Samsara”. The absolute of narcissus flowers has been widely used not only in popular classical perfumes in combination with rose, jasmine, violet and etc., but also in sophisticated modern perfumes, especially for strong pungent, green, woody and deep floral scent, which blends well with many other floral absolutes, such as ylang-ylang, jasmine, neroli, mimosa, clove bud carnation and others [2,11].

Industrially the absolutes are produced by extraction with organic solvent (hexane or other), its evaporation (causing some loss of volatile compounds), and removal of waxes. The distillation of fresh daffodil flowers recovers pure fraction of volatiles (essential oil), which are free of larger molecular weight waxes. However, the yields of EO are very low; for example, only 0.05% [12], while there is a risk of thermal degradation of some volatile compounds [11]. Nowadays green technologies for the isolation of valuable substances from plant materials are highly preferable in terms of safety and environmental protection; thus, instead of traditional distillation and/or solid-liquid extraction methods, the supercritical fluid extraction with carbon dioxide (SFE-CO_2_) was applied in our study for the recovery of lipophilic fraction containing fragrant volatile constituents. SFE-CO_2_ has been widely used for the recovery of EO together with other lipophilic constituents [13]. SFE-CO_2_ possesses many advantages (nontoxic, non-flammable, inexpensive, extraction at low temperatures avoids thermal degradation of the compounds and yields high purity extracts) and therefore can be successfully explored in pharmaceutical, cosmetics, food and other industries for recovery of valuable lipophilic constituents. On the other hand, the disadvantage of SFE-CO_2_ in the isolation of EO is that the extract also contains non-volatile lipophilic constituents.

More than 430 volatile compounds have been reported in *Narcissus* spp. [12,14,15,16,17,18,19]. For instance, 103 and 66 compounds were identified in *N. trevithian* and *N. geranium* oil, respectively, while only 20 of them were common for both species [17]. In the plants from Greece *(E)*-ocimene (61.12%) was dominant for *N. tazetta* and benzyl acetate (19.36%) for *N. serotinus* among 19 identified in the EOs constituents [12]. The main volatiles of *N. tazetta* absolute from Italy were γ-terpinene (27.21%), methyl cinnamate (15.84%), benzyl acetate (9.58%), benzyl alcohol (4.79%) and benzyl benzoate (4.00%) [18]. Eleven compounds were identified in the headspace of narcissus flowers; benzyl acetate (44.0%), 3,4- and 3,5-dimethoxy toluene (10.0 and 25.0%), and indole (5.0%) were major in the living flowers, whereas the percentages of the same compounds in the picked flowers were 30–43%, 18–39.5% and 0.3–1.0% [15]. *(E)*-β-Ocimene was found in high percentages in six species of *Narcissus* grown in Spain [19]. The main constituents of the Chinese narcissus flowers were *(E)*-β-ocimene (62.73–66.06%), benzyl acetate (11.65–25.02%), *(Z)*-β-ocimene, 1,8-cineole, and linalool [20].

Volatile compounds of *N. poeticus* have also been studied [2,11,21,22]. Ehret et al. [22] identified 80 new minor volatile components in *N. poeticus* absolute from France “Massif central”, α-terpineol (23.7%), methyl *(E)*-isoeugenol (20.0%), and benzyl benzoate (19.4%) being the major ones; more than 20 of them were considered as responsible for the complex floral notes reminiscent of “rose”, “jasmine”, “violet”, “tuberose” and “orange flower”. Cinnamyl alcohol (29.91 μg/g), methyl isoeugenol (28.07 μg/g), isoeugenol (23.12 μg/g), methyl eugenol (20.72 μg/g), and α-terpineol (20.31 μg/g) were the main components in hexane extract of fresh *N. poeticus* flowers of Rocca di Mezzo [11], whereas cinnamyl alcohol (30.2 μg/g), benzyl benzoate (28.5 μg/g), isoeugenol methyl ether (28.1 μg/g), *(Z)*-ocimene (25.2 μg/g), isoeugenol (22.9 μg/g) were quantitatively major in the plants from Sirente-Velino (Abruzzo region, Italy) [2]. To the best of our knowledge, only one article is available on SFE-CO_2_ of *N. poeticus* [11]; however, this study applied comparatively low pressure of the fluid CO_2_ (12 MPa). So far, as the solubility of many compounds increases with increasing solvent density, which depends on its pressure, in our study we applied CO_2_ pressure up to 48 MPa. It is important to mention that some of these volatiles are stated as more or less allergenic compounds, for example, cinnamyl alcohol, isoeugenol and cinnamyl aldehyde, and their use must follow legislation. It was suggested that in the preparation of safer perfumes absolute can be obtained from the corona only instead of tepals plus corona [2].

Considering previously reported data, our study aimed at evaluating the effects of pressure and addition of co-solvent in SFE-CO_2_ of freeze-dried *N. poeticus* flowers. Extract yields and comprehensive analysis of the recovered volatiles compounds were performed for this purpose. It is expected that the data obtained will expand our knowledge on the composition of *N. poeticus* fragrant constituents and the possibilities of their recovery by using green extraction techniques.

## 2. Results and Discussion

### 2.1. Extract Yields

The yields of the SFE-CO_2_ extracts of *N. poeticus* freeze-dried flowers varied from 1.63 ± 0.29 to 3.12 ± 0.12% (Table 1). The temperature of 40 °C, static extraction time 10 min and dynamic extraction time of 120 min were kept constant in all experiments. According to the literature reports, the yields of the concrete (absolute + waxes) obtained with hexane extraction were 0.2–0.3% [22], 0.41–0.45% [2,11] of fresh flowers and with petroleum ether –0.2% of fresh flowers [23]. The percentages of absolute in the concretes varied from 27 to 37% [11,23].

There were no statistical differences in the extract yields at 12–36 MPa, while at 48 MPa the yield of the recovered fraction was significantly higher (*p* < 0.05). Additionally, 5% ethanol applied as a co-solvent increased extract yield by ~32% (from 2.36 to 3.12%) compared with pure SFE-CO_2_ at 48 MPa. Ferri et al. [11] compared conventional hexane extraction with SFE-CO_2_ of *N. poeticus*; however, the absolute yields of SFE-CO_2_ extracts are not available in their article. Nevertheless, it is evident that SFE-CO_2_ extracts contained all the main constituents, which were identified in hexane extracts, while their yields in SFE-CO_2_ were significantly lower, even in case of using 5% ethanol, which enhanced the yields of most characteristics compounds [11].

### 2.2. Composition of N. poeticus SFE-CO_2_ Extracts

The detailed list of extracted by SFE-CO_2_ volatile compounds, their percentage composition and odour descriptions are presented in Table 2. In total, 116 compounds were identified in the SFE-CO_2_ extracts of *N. poeticus* flowers obtained under different extraction conditions. The sums (in %) and the numbers of the identified compounds in SFE-CO_2_ extracts at 12 MPa, 24 MPa, 36 MPa, 48 MPa and 48 MPa/EtOH were 77.47/105, 74.12/100, 76.07/104, 74.10/93, and 76.69/92, respectively.

It is evident that the composition of *N. poeticus* SFE-CO_2_ extracts is very complex, therefore for further discussion all the identified compounds were divided into 20 different classes, namely aromatics, aliphatic hydrocarbons, aromatic hydrocarbons, monoterpene hydrocarbons, oxygenated monoterpenes, oxygenated sesquiterpenes, esters, alcohols, aldehydes, ketones, lactones, acids, amides, diterpenoids, triterpenoids, tocopherols, phenylpropanoids, phenols and others (including oxanes, heteroaromatics, etc) (Table 2).

#### 2.2.1. Aromatics

Aromatic compounds is the most important group of volatiles in *N. poeticus* SFE-CO_2_ extracts; their percentage content varied from 12.55% (24 MPa) to 14.32% (48 MPa/EtOH). The major compounds were benzyl benzoate (65, 9.44–10.22%), benzyl linoleate (107, 1.72–2.17%), benzyl alcohol (12, 0.18–1.00%) and benzyl linolenate (108, 0.26–0.53%). Other aromatic compounds include hydrocinnamyl alcohol (30), 2-phenylethyl benzoate (70), benzyl 4-methoxybenzoate (75), benzoic acid hexadecyl ester (104), benzoic acid (21), benzyl oleate (106) (<0.1–0.2%), while hexyl benzoate (60) and *(3Z)*-hexenyl benzoate (58) were detected in trace amounts (Table 2).

**Figure 1:**
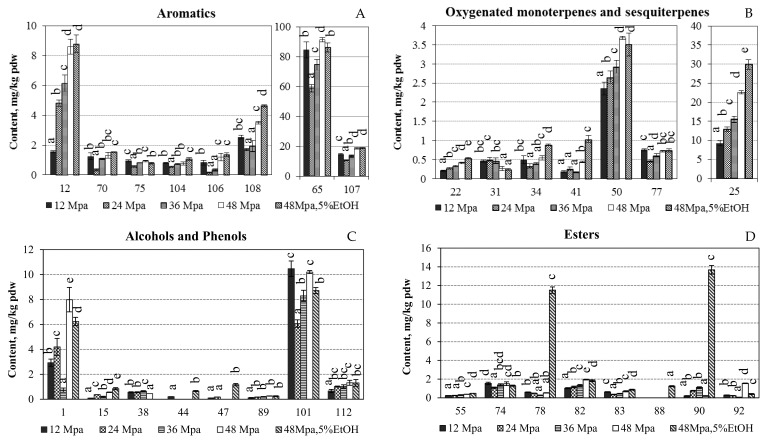
The absolute amounts of the main volatile compounds of major classes ((**A**)—Aromatics, (**B**)—Oxygenated monoterpenes and sesquiterpenes, (**C**)—Alcohols and phenols, (**D**)—Esters) of *Narcissus poeticus* SFE-CO_2_ extracts, in mg/kg pdw. Constituents are numbered by the same order as provided in Table 2. Values within columns followed by the same letter (a–e) do not differ statistically at *p* < 0.05 (Duncan test).

**Figure 2:**
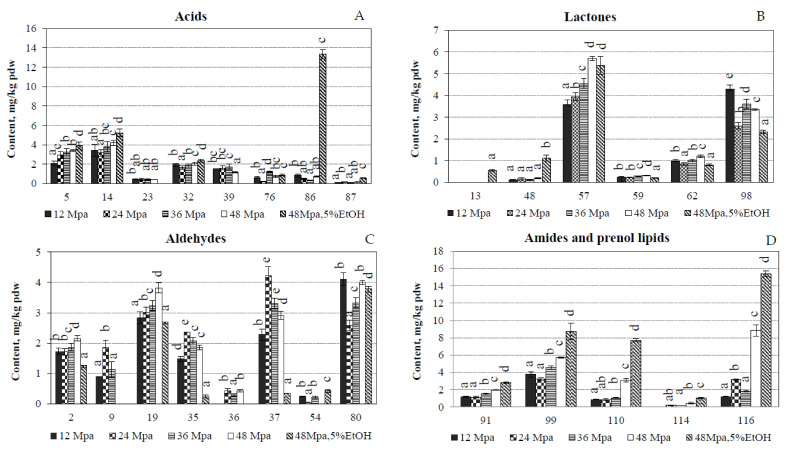
The absolute amounts of main volatile compounds of major classes ((**A**)—Acids, (**B**)—Lactones, (**C**)—Aldehydes, (**D**)—Amides and prenol lipids) of *Narcissus poeticus* SFE-CO_2_ extracts, in mg/kg pdw. Constituents are numbered by the same order as provided in Table 2. Values within columns followed by the same letter (a–e) do not differ statistically at *p* < 0.05 (Duncan test).

Benzyl benzoate, benzyl alcohol and benzoic acid are used in a wide variety of cosmetics formulations as fragrance ingredients and preservatives. The group of benzyl derivatives was reaffirmed as generally recognized as safe (GRAS) by the Expert Panel of the Flavour and Extract Manufacturers (FEMA), and the evidence of safety is supported by the fact that the intake of benzyl derivatives as natural components of traditional foods is larger than their intake in the case of intentional adding as flavouring substances [24]. Benzyl alcohol is an aromatic alcohol, which has been characterised as possessing “sweet, flower” [25] and “berry, cherry, grapefruit, citrus, and walnut” [26] odour notes. The absolute amounts of main volatile compounds in mg/kg pdw are summarised in Figure 1 and Figure 2. The highest recoveries of benzyl alcohol (12, 8.79 ± 0.59 mg/kg pdw), as well as benzyl linoleate (107, 18.96 ± 0.42 mg kg pdw) were obtained at 48 MPa/EtOH; however this value was not significantly different to the one obtained at 48 MPa (Figure 1A). Benzyl benzoate and benzyl alcohol were previously reported in high amounts in the absolutes of hexane extracts of *N. poeticus* from Italy [2,11].

It was reported that narcissus absolute has “a very strong, green, earthy and woody” odour, and that in appropriate dilution, releases a characteristic blend of floral and balsamic notes: light floral notes (rose or jasmine), deep floral notes (ylang-ylang, tuberose, orris and violet), notes of the balsamic type (styrax) and woody, earthy scents (oakmoss or patchouli) [22]. The balsamic type (styrax) flavour notes can be associated with the presence of benzoates and cinnamates in the *N. poeticus* SFE-CO_2_ products composition. Benzyl benzoate also possesses “balsamic, oil, herb” [25] and “almond, cheese, cherry, floral, pineapple, strawberry, sweet” [26] aroma notes. The absolute amount of the recovered benzyl benzoate from *N. poeticus* by SFE-CO_2_ varied from 58.98 ± 2.61 mg/kg pdw (24 MPa) to 91.52 ± 1.36 mg/kg pdw (48 MPa) (Figure 1A).

It was also reported that benzyl benzoate, its derivatives and benzyl cinnamate may be promising compounds in reducing hypertension [28]. It may be concluded that *N. poeticus* SFE-CO_2_ extracts after the separation of waxes might be a potential natural source of benzyl benzoate and benzyl alcohol.

#### 2.2.2. Monoterpene Hydrocarbons, Oxygenated Monoterpenes and Sesquiterpenes

α-Terpineol (25), which possess “oil, anise, mint” [25] and “lilac” [26] aroma notes, was major constituent among oxygenated monoterpenes (1.08–3.42%); it was also reported previously in *N. poeticus* absolute in comparatively high percentages [2,11,22]. The absolute amount of the recovered α-terpineol in *N. poeticus* SFE-CO_2_ extracts increased by increasing pressure and varied from 9.25 ± 0.63 mg/kg pdw (12 MPa) to 29.88 ± 1.25 mg/kg pdw (48 MPa/EtOH) (Figure 1B). It is well known that the quantitatively minor and even trace components can be important on the overall odour quality of flavours and fragrances. Consequently, the presence of ketones β-ionone epoxyde (0.28–0.42%) and trace component β-ionone may be important even at low concentrations (≤0.04%) by providing “violet flower aroma notes” of narcissus.

Limonene was the major monoterpene hydrocarbon; however, its percentage was low (0.08–0.17%). It is interesting that *(E)*-β-ocimene was not found in SFE-CO_2_ extracts of *N. poeticus* from Lithuania, while this compound was reported to be present in six species of *Narcissus* from Spain [19], *N. tazetta* EOs from Greece and *N. poeticus* absolute from Italy [2].

#### 2.2.3. Alcohols, Phenols, Lactones, Aldehydes and Other Volatiles

Among alcohols and phenols *(3E)*-hexenol (1) and heneicosanol (101) were recovered at the highest amounts (Figure 1C). *(3E)*-Hexenol is very abundant in various aromatic and spicy plants and possesses “moss” and “fresh” aroma notes. Therefore it may provide “green, woody” scents for narcissus even at low percentage concentrations, which varied from 0.10 ± 0.02% (36 MPa) to 0.89 ± 0.06% (48 MPa) (Table 2). The recovery of long-chain alcohol heneicosanol from *N. poeticus* by SFE-CO_2_ varied from 6.07 ± 0.30 mg/kg pdw (24 MPa) to 10.47 ± 0.62 mg/kg pdw (12 MPa) (Figure 1C).

The recoveries of esters (Figure 1D) and acids (Figure 2A) were quite low; however, it is interesting to mention that the amounts extracted from *N. poeticus* ethyl hexadecanoate (78), ethyl linolenate (90) and linoleic acid (86) significantly increased at 48 MPa and adding 5% of EtOH. It is believed that high amount of alcohols and long-chain acids in narcissus has more effect on the longer lasting trait of its odour, rather than on the odour quality [17].

Dihydroactinidiolide (57, 0.42–0.64%) and 4,8,12,16-tetramethyl heptadecan-4-olide (98, 0.27–0.50%) were the most abundant lactones in the SFE-CO_2_ extracts of *N. poeticus* (Figure 2B). The absolute content of dihydroactinidiolide was from 3.57 ± 0.23 mg/kg pdw (12 MPa) a to 5.70 ± 0.10 mg/kg pdw (48 MPa). Dihydroactinidiolide is a lactone (cyclic ester) resulting from the secondary oxidation of β-ionone through the intermediate 5,6-epoxy-β-ionone [29]. It possesses a “sweet, tea-like” odour and is used as a fragrance. Dihydroactinidiolide occurs naturally in plant leaves, black tea, fenugreek, fire ants, fruits, tobacco and it is a pheromone for a variety of insects [30]. Dihydroactinidiolide was reported to accumulate in Arabidopsis leaves under high light stress [31] and to exhibit cytotoxic effects against cancer cell lines [32].

Heptanal (2), nonanal (19), *(2E,4E)*-decadienal (37) and octadecanal (80) were the most abundant aldehydes (Figure 2C). Nonanal possesses very complex odour (Table 2) including “green” and “rose” scents. Its content in the extracts was 0.31–0.48%, while the absolute amounts, which were recovered from the flowers, varied from 2.70 ± 0.06 mg/kg pdw (48 MPa/EtOH) to 3.82 ± 0.16 mg/kg pdw (48 MPa) (Figure 2C). The amount of octadecanal was from 2.58 ± 0.18 mg/kg pdw (24 MPa) to 4.10 ± 0.22 mg/kg pdw (12 MPa). Octadecanal (or stearyl aldehyde) is a long chain fatty aldehyde and was identified as a biologically active pheromone component. Octadecanal is often used as the substrate of choice to test the microsomal enzyme fatty aldehyde dehydrogenase activity in patients suspected of having Sjogren-Larsson syndrome (autosomal recessively inherited neurocutaneous disorder) [33].

#### 2.2.4. Triterpenoids, Tocopherols and Others

Supercritical CO_2_ together with volatile compounds also extracts higher molecular weight lipophilic compounds, among them important prenol lipids such as triterpenoid squalene and tocopherols, which are well known antioxidants. Squalene is a long chain triterpene hydrocarbon, which is a precursor in the synthesis of sterols. The amount of squalene varied from 0.86 ± 0.10 mg/kg pdw (24 MPa) to 7.73 ± 0.18 mg/kg pdw (48 MPa/EtOH). It is worth noting that the use of a co-solvent ethanol increased the amount of extracted squalene ~2.5 times (Figure 2D). The antioxidant and oxygen carrying properties of squalene predicts its potential use in preventing cardiovascular diseases and cancer [34].

The amount of the recovered vitamin E (D-α-tocopherol) was from 1.20 ± 0.04 mg/kg pdw (12 MPa) to 15.39 ± 0.31 mg/kg pdw (48 MPa/EtOH). In nature, there are four main structures of tocopherols, namely α-, β-, γ-, and δ-tocopherol. Tocopherols are lipophilic molecules which are synthesized by plant cells and stored in leaves and seeds, and are endowed with antioxidant functions. They possess multiple beneficial healthy effects, such as the prevention of cardiovascular diseases and cancer [35].

The amide of oleic acid (Z)-9-Octadecenamide (99) and amide of palmitic acid–hexadecanamide (91), which are fatty acid derivatives, were determined in the lipophilic fraction. The percentage of *(Z)*-9-octadecenamide (99) was from 0.44 to 1.00% (Table 2) and the absolute amount *(Z)*-9-octadecenamide varied from 3.25 ± 0.13 mg/kg pdw (24 MPa) to 8.76 ± 0.96 mg/kg pdw (48 MPa/EtOH) (Figure 2D). Oleamide was reported in various natural plant materials such as *Nigella sativa* [36], mountain celery seeds [37] and other plants [38], however it should be noted that it may be an artefact, which is transferred into the extracts from the labware [39]. The health-promoting properties of *(Z)*-9-octadecenamide have been reported for this oleamide, such as anti-inflammatory and antibacterial activities [36], hypolipidemic activity [37], against Alzheimer disease [38], and etc.

Some of the components depending on different classes were identified in narcissus extract when the highest pressure and addition of 5% ethanol as co-solvent were applied, e.g., pantolactone (13), ethyl heptanoate (17), benzoic acid (21), *(E)*-8-hydroxylinalool (40), 3-hydroxydecanoic acid (56), ethyl tetradecanoate (66), ethyl 9-octadecenoate (88).

#### 2.2.5. Aliphatic Hydrocarbons (Waxes)

It was observed that the saturated hydrocarbons (n-alkanes) constitute a very large fraction in the total amount (from 41.47 to 54.93%) of GC-detectable volatiles of the narcissus lipophilic fraction (SFE-CO_2_ extracts). The percentage of *n*-alkanes slightly decreased by increasing pressure; it was expected additionally that by applying 5% ethanol as a co-solvent the percentage amount of alkanes was the lowest (41.47%). Higher *n*-alkanes, such as heptacosane (105, 11.21–15.40%), tricosane (96, 7.57–11.36%), nonacosane (111, 5.64–7.27%), untriacontane (115, 4.41–6.73%), pentacosane (102, 4.18–6.13%) and heneicosane (81, 3.71–5.50%) were the major qualitative compounds in this fraction (Table 2).

**Figure 3:**
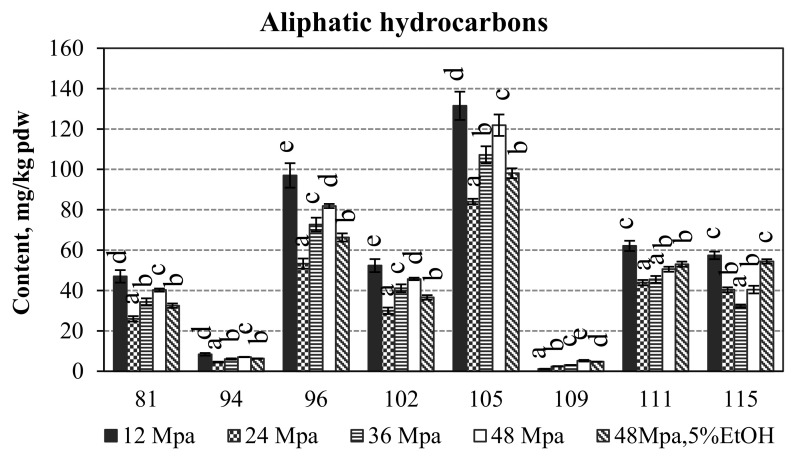
The absolute amounts of aliphatic hydrocarbons in *Narcissus poeticus* SFE-CO_2_ extracts, in mg/kg pdw. Constituents are numbered by the same order as provided in Table 2. Values within columns followed by the same letter (a–e) do not differ statistically at *p* < 0.05 (Duncan test).

The highest absolute amounts of aliphatic hydrocarbons were extracted at 12 MPa, while the lowest at 24 MPa. Further increase in pressure from 24 to 48 MPa resulted in the increase in the recovered amounts of *n*-alkanes, while in the case of using ethanol it was reduced again. For example, the highest amount of the recovered *n*-heptacosane (105) was 131.49 ± 7.00 mg/kg pdw at 12 MPa and the lowest 84.07 ± 1.37 mg/kg pdw at 24 MPa (Figure 3). At further increase in pressure its amount increased to 121.88 ± 5.30 mg/kg pdw (48 MPa), while 5% ethanol reduced the value to 98.08 ± 5.30 mg/kg pdw, which was significantly lower to that extracted at 12 MPa. The amount of these saturated acyclic alkanes could be reduced by using conventional dewaxing procedures.

## 3. Materials and Methods

### 3.1. Plant Material and Chemicals

*Narcissus poeticus* L. plants were grown in a farmstead near the city of Klaipėda (Lithuania, coordinates: 55°45′ N 21°10′ E) and the flowers were collected at a full flowering stage. The flowers were picked in the morning manually, separated from the stems by scissors. Fresh flowers were frozen to −40 °C and freeze-dried in a Sublimator 40 at 0.05 mbar pressure (Zirbus Technology, Bad Grund, Germany). The dried flowers were ground in an ultra-centrifugal mill ZM 200 (Retsch, Haan, Germany) using 0.5 mm hole size sieve.

Pentane (for residue analysis, ≥99.0%) was from Sigma-Aldrich (Steinheim, Germany), Ethanol (≥96%) obtained from Joint Stock Company Stumbras, Lithuania. C_7_–C_30_ saturated *n*-alkanes standard and internal standard (decane) were from Supelco Analytical (Bellefonte, PA, USA). The following reference compounds (95–99% purity) for the identification of narcissus volatiles were purchased from Fluka, Sigma–Aldrich or Supelco: limonene, *p*-cymene, α-terpineol, vanillin, benzyl alcohol, nonanal, β-ionone, caryophyllene oxide, squalene.

### 3.2. Supercritical Carbon Dioxide Extraction (SFE-CO_2_)

SFE-CO_2_ was carried out in a supercritical fluid extractor Helix (Applied Separation, Allentown, PA, USA). Each extraction was performed from 10 g of ground *N. poeticus* flowers placed in a 50 mL cylindrical extractor vessel (14 mm × 320 mm; h/d = 22.86), between two layers of defatted cotton wool in both ends, to avoid particles clogging the system. The temperature of the extraction vessel was controlled by a surrounding heating jacket. The flow rate of CO_2_ in the system (v) was controlled manually by the micro-metering valve (back-pressure regulator). The volume of CO_2_ consumed was measured by a ball float rotameter and a digital mass flow meter in liters per min (SL/min) at standard state: pressure P = 100 kPa, temperature T = 20 °C, density ρ = 0.0018 g/mL. The conditions for extraction were set as follows (see in Table 1): extraction time 120 min, pressure 12–48 MPa, extraction temperature 40 °C, flow rate of CO_2_ 2 L/min. A static time of 10 min was included in to the total extraction time and was constant. The extraction at 48 Mpa was also performed using 5% (*v*/*v*) ethanol as a co-solvent in order to enhance the polarity of the solvent mixture. The extracts were collected in brown glass bottles at room temperature and atmospheric pressure and stored at −20 °C until further analysis.

### 3.3. Gas Chromatography–Time-of-Flight Mass Spectrometry (GC-TOF/MS)

For qualitative and quantitative composition of SFE-CO_2_ extracts of *N. poeticus* flowers, they diluted in pentane (10 mg/mL) containing 0.2% internal standard and analysed on a GC × GC-TOFMS LECO Pegasus 4D system, consisting of an Agilent 7890A GC, a GERSTEL Multipurpose Sampler MPS (Gerstel GmbH, Mulheim an der Ruhr, Germany), a high-speed TOF/MS detector (LECO, St. Joseph, MI, USA) and a four jet cryogenic modulator (Zoex, Houston, TX, USA) by comparing the 1D first dimension linear temperature programmed Kováts retention index with the peak identities provided by a mass spectral similarity search. The column set consisted of a primary column BPX-5 (30 m, 0.25 mm i.d., 0.25 μm film thickness) (SGE Analytical Science, Australia) connected to a secondary column, BPX-50 (1.8 m, 0.10 mm i.d., 0.1 μm film thickness). The primary oven was programmed as follows: 50 °C ramped to 100 °C at 10 °C/min (0 min) further ramped to 300 °C at 5 °C/min (hold 5 min); the secondary oven programming was from 65 °C ramped to 115 °C at 10 °C/min (0 min) then ramped to 315 °C at 5°C/min (hold 5 min). The transfer line temperature was 250 °C, the GC injector port was kept at 280 °C with desorption time of 5 min.

The TOF/MS acquisition rate was 10 spectra/s, the mass range used for identification was from 35–550 m/z units. Detector’s voltage was set at 1550 V and ion source temperature of 250 °C. Data from the GC × GC-TOF/MS system were collected by ChromaTOF software v.4.22 (LECO) after a solvent peak delay of 420 s in a splitless mode for 30 s, a further valve was opened and purge flow was 20 mL/min; mass spectrum assignment was based on matching against Adams, NIST, MainLib, RepLib mass spectra libraries; signal-to-noise threshold was set as 50 with the minimum similarity accepted was 700. The mean values were calculated from triplicate injections.

Quantitative data were obtained by peak area normalization without using correction factors as the means of triplicate GC-TOF/MS runs and expressed as peak area percentage and recalculated in mg/kg per plant dry weight (mg/kg pdw). Quantitative data were calculated using the formula: $m_{i}=\left(m_{D}\times A_{i}/A_{D}\times RF_{i}\right)\times \left(\left(Y_{e}\times 1000/100\right)/m_{e}\right)$, where $m_{i}$ is the mass of the individual compound *i* to be quantified, expressed in mg/kg per plant dry weight (mg/kg pdw); $m_{D}$ mass of decane (internal standard, IS), $A_{i}$ and $A_{D}$—are the peak area of the analyte *i* and the IS, respectively, $RF_{i}$—response factor of individual compound; $Y_{e}$—the yield of the extract (%), $m_{e}$—the mass of the extract (g).

The identification of volatile components was assigned by comparing their Kováts Retention Indices (KI) relative to C_7_–C_30_ *n*-alkanes, obtained on nonpolar BPX-5 column with those provided in literature [27] and by comparing their mass spectra with the data provided by the NIST, MainLib, RepLib and Adams mass spectral libraries. The identity of some constituents was confirmed by co-injection of reference compounds. Positive identification was assumed when good match of mass spectrum and KI was achieved.

### 3.4. Statistical Analysis

All analyses were replicated at least three times and all data are reported as mean values ± standard deviations (SD) using MS Excel 2010 software. Data were statistically handled by one-way analysis of variance (ANOVA, vers. 2.2), significant differences among the samples were evaluated by the Duncan’s multiple-range test at the probability level of *p* < 0.05.

## 4. Conclusions

*Narcissus poeticus* flowers were extracted with supercritical carbon dioxide at different solvent pressures, from 12 to 48 MPa. The yield of lipophilic fraction significantly increased after raising the pressure from 36 to 48 MPa and by adding 5% of a co-solvent ethanol into the CO_2_ flow. Generally, the yields were higher compared with the previously reported data for conventional extraction with organic solvents. In total, 116 volatile compounds were identified by GC-TOF/MS in the extracts. The most important for *N. poeticus* odour constituents benzyl benzoate (9.44–10.22%), benzyl linoleate (1.72–2.17%) and benzyl alcohol (0.18–1.00%) were the major volatiles among aromatic compounds. The extracts contained a large fraction of waxes, which are not desirable in the production of higher quality fragrance ingredients; however, adding a co-solvents ethanol enabled the reduction in the percentage of higher alkanes, while the amount of the recovered benzyl aromatics increased. On the other hand, for practical purposes, SFE-CO_2_ at 48 MPa would most likely be preferable because in this case the process becomes less complicated and cheaper; there is no need for a co-solvent pump and removal of it after extraction, which may result in some loss of volatile fragrance constituents. However, adding co-solvent increases the recovery of an important bioactive compound α-tocopherol approx. 2-fold. In general, the results obtained are promising for a wider application of supercritical extraction for the recovery of fragrance substances from *Narcissus poeticus* flowers.

## Figures and Tables

**Table 1:** SFE-CO_2_ extraction conditions of *Narcissus poeticus* and obtained yields (%).

SFE-CO_2_ Extraction Conditions	Extract Yield, %
12 MPa	1.63 ± 0.29 ^a^
24 MPa	1.75 ± 0.10 ^a^
36 MPa	1.82 ± 0.09 ^a^
48 MPa	2.36 ± 0.09 ^b^
48 MPa + 5% EtOH	3.12 ± 0.12 ^c^

Results are expressed as a mean ± standard deviation (*n* = 3); Values within columns followed by the same letter (a–c) do not differ statistically at *p* < 0.05 (Duncan test).

**Table 2:** Chemical composition of *Narcissus poeticus* SFE-CO_2_ extracts, peak area percentage (%).

No.#	Compound ^A^	KI Calc. ^B^	KI Lit. ^C^	KI Lit. ^D^	12 Mpa	24 Mpa	36 Mpa	48 Mpa	48 Mpa/EtOH	Odour Description ^1^
1.	*(3E)*-Hexenol	854	853	1386	0.35 ± 0.02 ^c^	0.67 ± 0.10 ^b^	0.10 ± 0.02 ^a^	0.89 ± 0.06 ^d^	0.72 ± 0.06 ^b^	moss, fresh
2.	Heptanal	907	902	1174	0.20 ± 0.01 ^c^	0.27 ± 0.02 ^d^	0.26 ± 0.01 ^b^	0.24 ± 0.02 ^b^	0.15 ± 0.01 ^a^	fat, citrus, rancid ^1^; oily, fruity, woody, fatty, nutty ^2^
3.	tetrahydro-Citronellene	938	937		tr ^a^	0.05 ± 0.00 ^b^	tr ^a^	tr ^a^	0.07 ± 0.00 ^c^	
4.	2-methyl-Nonane	971	970 ^E^	961 ^F^	0.06 ± 0.00 ^b^	0.09 ± 0.01 ^e^	0.07 ± 0.01 ^c^	0.08 ± 0.00 ^d^	tr ^a^	
5.	Hexanoic acid	978	973	1829	0.24 ± 0.02 ^a^	0.47 ± 0.05 ^d^	0.45 ± 0.03 ^b^	0.39 ± 0.00 ^e^	0.45 ± 0.04 ^bcd^	sweat ^1^; cheese, fatty, sour ^2^
6.	2-Pentyl furan	992	988	1240	0.13 ± 0.00 ^a^	0.22 ± 0.01 ^d^	0.19 ± 0.01 ^c^	0.16 ± 0.01 ^b^		green bean, butter ^1^; green, vegetable ^2^
7.	Mesitylene	998	995	1220 ^E^	tr ^a^	tr ^a^	tr ^a^	0.18 ± 0.00 ^b^		
8.	Decane *	1000	1000	1000	0.12 ± 0.01 ^a^	0.15 ± 0.00 ^b^	0.17 ± 0.01 ^b^	0.20 ± 0.02 ^d^	0.20 ± 0.01 ^d^	alkane
9.	*(2E,4E)*-Heptadienal	1014	1007	1401	0.10 ± 0.00 ^a^	0.30 ± 0.04 ^c^	0.15 ± 0.03 ^b^			nut, fat ^1^; cinnamon, hazelnut, fatty ^2^
10.	*p*-Cymene	1025	1025	1261	tr ^a^	tr ^b^	tr ^a^	tr ^a^		solvent, gasoline, citrus
11.	Limonene	1028	1029	1178	0.12 ± 0.00 ^b^	0.17 ± 0.01 ^d^	0.15 ± 0.00 ^c^	0.11 ± 0.01 ^b^	0.08 ± 0.00 ^a^	lemon, orange ^1^; citrus, sweet ^2^
12.	Benzyl alcohol	1034	1031	1865	0.18 ± 0.01 ^a^	0.77 ± 0.04 ^b^	0.84 ± 0.05 ^b^	0.96 ± 0.01 ^c^	1.00 ± 0.02 ^c^	sweet, flower ^1^; berry, cherry, grapefruit, citrus, walnut ^2^
13.	Pantolactone	1037	1032 ^F^	2034 ^F^					0.06 ± 0.00	cotton candy
14.	Heptanoic acid	1078	1074 ^E^	1918 ^E^	0.40 ± 0.06 ^a^	0.51 ± 0.05 ^c^	0.52 ± 0.05 ^bc^	0.47 ± 0.06 ^b^	0.60 ± 0.02 ^d^	sour ^2^
15.	*p*-Cresol	1083	1076	2067	tr ^a^	0.06 ± 0.00 ^b^	tr ^c^	0.06 ± 0.00 ^b^	0.10 ± 0.01 ^d^	medicine, phenol, smoke ^1^; woody, ethereal, medicinal ^2^
16.	2-Nonanone	1097	1090	1388	tr ^a^	tr ^a^	tr ^a^			hot milk, soap, green ^1^; cheese, coconut, oily, fatty, herbaceous, floral, fruity, fishy, soapy, waxy ^2^
17.	Ethyl heptanoate	1102	1093 ^E^	1321 ^E^					tr	
18.	*(4E)*-Nonenal	1106	1096 ^G^	1458 ^E^	tr ^a^	tr ^b^	tr ^ab^			berry, melon, peach, pineapple, plum ^2^
19.	Nonanal	1109	1100	1385	0.33 ± 0.01 ^c^	0.48 ± 0.02 ^d^	0.44 ± 0.01 ^b^	0.43 ± 0.04 ^b^	0.31 ± 0.01 ^a^	fat, citrus, green ^1^; apple, coconut, grape, lemon, grapefruit, lime, melon, orange, nutty, citrus, oily, waxy, fatty, peach, rose, vegetable, fishy, meaty ^2^
20.	*(2E)*-Nonenal	1167	1161	1527		tr				cucumber, fat, green ^1^; waxy, fatty ^2^
21.	Benzoic acid	1163	1162 ^E^	1624					0.11 ± 0.02	urine ^1^; balsam ^2^
22.	δ-Terpineol	1170	1166	1655	tr ^a^	tr ^ab^	tr ^ab^	0.05 ± 0.00 ^b^	0.06 ± 0.00 ^c^	
23.	Octanoic acid	1183	1171	2083	0.05 ± 0.00 ^a^	0.07 ± 0.02 ^c^	0.06 ± 0.0 ^abc^	0.05 ± 0.00 ^a^		sweat, cheese ^1^; oily ^2^
24.	Naphthalene	1191	1181	1712 ^E^	tr ^ab^	tr ^ab^	tr ^b^			
25.	α-Terpineol	1194	1188	1688	1.08 ± 0.04 ^a^	2.07 ± 0.10 ^b^	2.12 ± 0.06 ^b^	2.52 ± 0.19 ^c^	3.42 ± 0.16 ^d^	oil, anise, mint ^1^; lilac ^2^
26.	*(2E)*-Hexenyl butanoate	1197	1194	1458 ^E^	tr ^a^	tr ^a^	0.08 ± 0.00 ^b^			apple, cheese, green, meaty ^2^
27.	*n*-Dodecane	1200	1200	1200		tr ^ab^	tr ^ab^	tr ^b^		alkane
28.	*exo*-2-Hydroxycineole	1219	1219 ^E^	1845 ^E^		tr ^ab^	tr ^ab^	tr ^a^	tr ^b^	
29.	2-Hydroxycineole	1230	1229 ^E^		0.06 ± 0.01 ^a^	0.11 ± 0.02 ^b^	0.08 ± 0.00 ^a^	0.08 ± 0.01 ^a^	0.11 ± 0.00 ^b^	
30.	Hydrocinnamyl alcohol	1242	1231 ^E^	1989 ^E^	tr ^b^	0.09 ± 0.00 ^c^	0.05 ± 0.00 ^b^	tr ^a^	0.09 ± 0.01 ^c^	balsam, hyacinth, floral, sweet ^2^
31.	Carvone	1249	1243	1720	0.05 ± 0.00 ^b^	0.08 ± 0.01 ^d^	0.06 ± 0.01 ^c^	tr ^a^	tr ^a^	caraway, mint, basil, fennel ^1^; herbaceous ^2^
32.	Nonanoic acid	1280	1270	2202	0.23 ± 0.01 ^a^	0.26 ± 0.03 ^bc^	0.26 ± 0.01 ^bc^	0.23 ± 0.03 ^a^	0.27 ± 0.02 ^c^	green, fat ^1^; cheese, waxy ^2^
33.	*n*-Tridecane	1301	1300	1300	tr ^a^	0.06 ± 0.00 ^bc^	0.06 ± 0.00 ^b^	0.06 ± 0.01 ^bc^	0.06 ± 0.00 ^c^	alkane
34	8-Hydroxymenthol	1304	1301	2167 ^E^	0.06 ± 0.01 ^a^	0.05 ± 0.01 ^a^	0.05 ± 0.00 ^a^	0.06 ± 0.01 ^a^	0.10 ± 0.01 ^b^	
35.	*(2E,4Z)*-Decadienal	1306	1303 ^E^	1710	0.17 ± 0.01 ^b^	0.44 ± 0.02 ^e^	0.28 ± 0.0 ^c^	0.21 ± 0.02 ^d^	tr ^a^	fried, fat
36.	Undecanal	1308	1306	2444		0.07 ± 0.01 ^b^	tr ^a^	0.05 ± 0.01 ^ab^		oil, pungent, sweet
37.	*(2E,4E)*-Decadienal	1317	1316	1710	0.27 ± 0.01 ^b^	0.68 ± 0.04 ^e^	0.45 ± 0.00 ^d^	0.32 ± 0.03 ^c^	tr ^a^	fried, wax, fat ^1^; fatty, citrus, meaty ^2^
38.	*(2E)*-Undecenol	1366	1365	1899 ^F^	0.07 ± 0.00 ^c^	0.09 ± 0.00 ^b^	0.09 ± 0.00 ^b^	0.05 ± 0.01 ^a^		
39.	Decanoic acid	1367	1366	2361	0.17 ± 0.00 ^b^	0.26 ± 0.03 ^d^	0.24 ± 0.02 ^c^	0.13 ± 0.01 ^a^		rancid, fat ^1^; fatty, citrus ^2^
40.	*(E)*-8-Hydroxylinalool	1370	1367	2265 ^E^					0.13 ± 0.00	
41.	*(E)*-*p*-Menth-6-en-2,8-diol	1377	1374	1740 ^E^	tr ^a^	tr ^ab^	tr ^a^	0.05 ± 0.00 ^b^	0.12 ± 0.01 ^c^	
42.	3-Dodecanone	1391	1390	1655 ^E^	0.05 ± 0.01 ^a^	0.05 ± 0.00 ^a^	0.06 ± 0.00 ^a^	0.06 ± 0.01 ^a^	0.11 ± 0.00 ^b^	
43.	*n*-Tetradecane	1400	1400	1400	tr ^c^	tr ^a^	tr ^b^			alkane
44.	Vanillin	1406	1407	2540 ^E^	tr ^a^				0.08 ± 0.01 ^b^	caramel, chocolate, sweet, vanilla ^2^
45.	Carvone hydrate	1432	1423	1754 ^E^	tr ^a^		tr ^a^	tr ^ab^	tr ^b^	
46.	1b,5,5,6a-Tetramethyl-octahydro-1-oxa-cyclopropa[a]inden-6-one	1454	1445 ^G^		tr ^a^	tr ^b^	0.11 ± 0.01 ^c^	tr ^ab^		
47.	*(E)*-Isoeugenol	1455	1451	2372	tr ^a^	tr ^b^			0.13 ± 0.01 ^c^	flower ^1^; clove, sweet, woody, spicy ^2^
48.	3a,4,7,7a-tetrahydro-3a-methyl-2(3H)-Benzofuranone	1457	1456 ^E^	2235 ^E^	tr ^a^	tr ^b^	tr ^ab^	tr ^ab^	0.13 ± 0.01 ^c^	
49.	*(E)*-β-Ionone	1489	1488	1912	tr ^ab^	tr ^c^	tr ^bc^	tr ^ab^	tr ^a^	seaweed, violet, flower, raspberry ^1^; almond, basam, berry, grape, jam, orange, fruity, woody, floral, peach, raspberry, rose, sweet, violet, minty, wine-like, vegetable ^2^
50.	β-Ionone epoxide	1494	1488 ^E^	1957 ^E^	0.28 ± 0.01 ^a^	0.42 ± 0.02 ^d^	0.40 ± 0.01 ^b^	0.41 ± 0.02 ^c^	0.40 ± 0.02 ^bc^	fruit, sweet, wood
51.	*(E)*-Methyl isoeugenol	1497	1492	2176 ^E^	tr ^a^	tr ^b^	tr ^b^	tr ^b^	0.05 ± 0.00 ^c^	spicy ^2^
52.	2-Tridecanone	1499	1496	1805 ^E^	tr ^a^	0.05 ± 0.00 ^b^	0.05 ± 0.00 ^b^	0.05 ± 0.00 ^b^	tr ^a^	spicy ^1^; herbaceous ^2^
53.	*n*-Pentadecane	1500	1500	1500	0.05 ± 0.01 ^b^	tr ^a^	0.07 ± 0.00 ^c^	0.06 ± 0.00 ^bc^	0.06 ± 0.00 ^bc^	alkane
54.	Tridecanal	1515	1510	1824	tr ^ab^	tr ^a^	tr ^ab^		0.05 ± 0.01 ^c^	flower, sweet, must
55.	Methyl dodecanoate	1530	1525	1795	tr ^a^	tr ^b^	tr ^b^	tr ^b^	0.06 ± 0.00^c^	fat, coconut ^1^; coconut, creamy, soapy, waxy ^2^
56.	3-Hydroxydecanoic acid	1536	1534						0.10 ± 0.01	
57.	Dihydroactinidiolide	1546	1535 ^E^	2308 ^E^	0.42 ± 0.01 ^a^	0.63 ± 0.03 ^d^	0.62 ± 0.01 ^bc^	0.64 ± 0.04 ^cd^	0.62 ± 0.02 ^b^	
58.	*(3Z)*-Hexenyl benzoate	1570	1566	2122 ^E^	0.05 ± 0.00 ^b^		tr ^a^		tr ^a^	woody, herbaceous, green ^2^
59.	γ-Undecalactone	1576	1570	2270	tr ^bc^	tr ^b^	tr ^b^	tr ^b^	tr ^a^	apricot ^1^; musty, peach, sweet, earthy ^2^
60.	Hexyl benzoate	1582	1580	2066 ^E^	tr					balsam, woody, green ^2^
61.	Caryophyllene oxide	1587	1583	1962	tr ^a^		tr ^b^			herb, sweet, spice
62.	γ-Dodecalactone	1687	1677	2384	0.12 ± 0.00 ^b^	0.13 ± 0.01 ^bc^	0.14 ± 0.00 ^c^	0.14 ± 0.01 ^bc^	0.09 ± 0.00 ^a^	fruit, sweet ^1^; musty, fatty, fruity ^2^
63.	2-Pentadecanone	1697	1697	2016 ^E^	0.17 ± 0.00 ^a^	0.17 ± 0.01 ^ab^	0.18 ± 0.00 ^ab^	0.18 ± 0.01 ^b^	0.16 ± 0.00 ^a^	
64.	Heptadecane	1700	1700	1700	tr ^b^	tr ^c^	tr ^b^	tr ^a^		alkane
65.	Benzyl benzoate	1763	1760	2071	9.90 ± 0.34 ^a^	9.44 ± 0.44 ^a^	10.22 ± 0.16 ^ab^	10.22 ± 0.69 ^b^	9.85 ± 0.12 ^a^	balsamic, oil, herb ^1^; almond, cheese, cherry, floral, pineapple, strawberry, sweet ^2^
66.	Ethyl tetradecanoate	1799	1796	2042					0.08 ± 0.00	ether ^1^; waxy, soapy ^2^
67.	Octadecane	1800	1800	1800	tr					alkane
68.	2-Hexadecanone	1816	1809	2112	0.06 ± 0.00 ^c^	0.06 ± 0.00 ^bc^	0.06 ± 0.00 ^bc^	0.05 ± 0.00 ^abc^	tr ^a^	fruit
69.	Hexadecanal	1828	1818 ^E^	2141 ^E^	tr ^a^	tr ^ab^	tr ^b^	tr ^b^		
70.	2-Phenylethyl benzoate	1865	1859 ^E^	2189	0.15 ± 0.02 ^bc^	0.05 ± 0.01 ^a^	0.15 ± 0.01 ^bc^	0.15 ± 0.02 ^bc^	0.18 ± 0.01 ^c^	flower, honey ^1^; honey, rose ^2^
71.	3-Heptadecanone	1884	1880 ^E^	2155 ^E^	0.05 ± 0.00 ^a^	0.05 ± 0.00 ^a^	0.05 ± 0.01 ^a^	0.05 ± 0.01 ^a^	0.20 ± 0.01 ^b^	
72.	*n*-Nonadecane	1900	1900	1900	0.20 ± 0.01 ^d^	0.17 ± 0.01 ^a^	0.19 ± 0.00 ^bcd^	0.19 ± 0.01 ^cd^	0.18 ± 0.00 ^ab^	alkane
73.	2-Heptadecanone	1904	1900 ^E^	2245 ^E^	tr ^a^	tr ^a^	tr ^a^	tr ^a^		
74.	Methyl hexadecanoate	1922	1921	2204 ^E^	0.18 ± 0.01 ^bc^	0.17 ± 0.01 ^bc^	0.19 ± 0.01 ^c^	0.17 ± 0.02 ^bc^	0.15 ± 0.00 ^a^	
75.	Benzyl 4-methoxybenzoate	1925	1922 ^G^		0.11 ± 0.00 ^c^	0.09 ± 0.01 ^a^	0.10 ± 0.00 ^b^	0.10 ± 0.01 ^b^	0.09 ± 0.00 ^a^	
76.	Hexadecanoic acid	1967	1960	2931 ^E^	0.07 ± 0.01 ^b^	tr ^a^	0.16 ± 0.02 ^c^	0.08 ± 0.02 ^b^	0.10 ± 0.01 ^b^	
77.	Geranyl benzoate	1982	1978		0.09 ± 0.00 ^c^	0.07 ± 0.00 ^a^	0.08 ± 0.00 ^bc^	0.08 ± 0.01 ^ab^	0.08 ± 0.00 ^bc^	
78.	Ethyl hexadecanoate	1997	1993	2250	0.07 ± 0.00 ^bc^	0.08 ± 0.00 ^c^	0.04 ± 0.00 ^a^	0.06 ± 0.00 ^b^	1.31 ± 0.02 ^d^	waxy
79.	*n*-Eicosane	2000	2000	2000	0.05 ± 0.00					alkane
80.	Octadecanal	2036	2033 ^E^	2400	0.48 ± 0.01 ^c^	0.41 ± 0.03 ^a^	0.46 ± 0.00 ^b^	0.45 ± 0.03 ^b^	0.43 ± 0.01 ^ab^	oil
81.	*n*-Heneicosane	2100	2100	2100	5.50 ± 0.19 ^d^	4.16 ± 0.22 ^b^	4.69 ± 0.08 ^c^	4.49 ± 0.31 ^c^	3.71 ± 0.06 ^a^	alkane
82.	Methyl oleate	2105	2103	2430	0.12 ± 0.00 ^a^	0.19 ± 0.01 ^b^	0.18 ± 0.01 ^b^	0.22 ± 0.01 ^d^	0.21 ± 0.00 ^c^	fat
83.	Methyl linolenate	2113	2108 ^E^	2590 ^E^	0.07 ± 0.00 ^b^	0.06 ± 0.00 ^a^	0.06 ± 0.00 ^a^	0.08 ± 0.00 ^c^	0.10 ± 0.00 ^d^	
84.	Phytol	2115	2115 ^E^	2571	0.05 ± 0.00 ^ab^	tr ^a^	tr ^a^	0.06 ± 0.01 ^b^	0.23 ± 0.00 ^c^	flower ^1^; balsamic, floral ^2^
85.	*(E)*-Benzyl cinnamate	2131	2134 ^E^	2769 ^E^	tr ^a^		tr ^b^			apricot, cherry, chocolate, floral, peach, pineapple ^2^
86.	Linoleic acid	2139	2134 ^E^	3168 ^E^	0.10 ± 0.01 ^c^	0.08 ± 0.01 ^b^	0.05 ± 0.00 ^a^	0.08 ± 0.00 ^bc^	1.53 ± 0.02 ^d^	
87.	Oleic acid	2147	2142	2430	tr ^a^	tr ^b^	tr ^a^	tr ^a^	0.07 ± 0.00 ^c^	fat
88.	Ethyl 9-octadecenoate	2162	2150 ^E^	2469 ^E^					0.14 ± 0.00	
89.	*n*-Nonadecanol-1	2171	2156 ^E^	2637 ^E^	tr ^a^	tr ^b^	tr ^b^	tr ^b^	tr ^b^	
90.	Ethyl linolenate	2176	2169 ^E^	2621 ^E^	tr ^a^	0.12 ± 0.01 ^b^	0.15 ± 0.00 ^b^	tr ^a^	1.56 ± 0.03 ^c^	
91.	Hexadecanamide	2189	2182 ^E^	2858 ^E^	0.14 ± 0.01 ^a^	0.18 ± 0.01 ^b^	0.20 ± 0.00 ^c^	0.22 ± 0.01 ^d^	0.32 ± 0.01 ^e^	
92.	*n*-Butyl hexadecanoate	2194	2188 ^E^	2419 ^E^	tr ^b^	tr ^c^	tr ^a^	0.17 ± 0.01 ^d^	0.05 ± 0.01 ^c^	
93.	1-Docosene	2199	2189		0.10 ± 0.00 ^c^	0.09 ± 0.00 ^b^	0.10 ± 0.00 ^c^	0.10 ± 0.01 ^c^	0.07 ± 0.00 ^a^	
94.	*n*-Docosane	2200	2200	2200	0.98 ± 0.05 ^d^	0.72 ± 0.04 ^ab^	0.83 ± 0.02 ^c^	0.79 ± 0.05 ^bc^	0.71 ± 0.02 ^a^	alkane
95.	Tributyl acetylcitrate	2258	2250 ^E^		tr ^a^		tr ^a^			
96.	*n*-Tricosane	2300	2300	2300	11.36 ± 0.39 ^e^	8.54 ± 0.44 ^b^	9.94 ± 0.17 ^d^	9.14 ± 0.55 ^c^	7.57 ± 0.12 ^a^	alkane
97.	2-Ethylhexyl *p*-methoxy cinnamate	2349	2339 ^F^	3122 ^F^	tr ^b^	tr ^ab^	tr ^b^	tr ^b^	tr ^b^	
98.	4,8,12,16-Tetramethyl heptadecan-4-olide	2364	2364 ^E^		0.50 ± 0.01 ^d^	0.42 ± 0.02 ^c^	0.49 ± 0.01 ^d^	0.38 ± 0.02 ^b^	0.27 ± 0.00 ^a^	
99.	*(Z)*-9-Octadecenamide	2398	2397 ^E^	3265 ^E^	0.44 ± 0.04 ^a^	0.52 ± 0.02 ^b^	0.62 ± 0.01 ^c^	0.64 ± 0.03 ^c^	1.00 ± 0.06 ^d^	
100.	*n*-Tetracosane	2400	2400	2400	0.27 ± 0.01 ^a^	0.37 ± 0.01 ^c^	0.43 ± 0.01 ^e^	0.40 ± 0.02 ^d^	0.35 ± 0.00 ^b^	alkane
101.	Heneicosanol	2403	2402 ^E^	2995 ^E^	1.23 ± 0.04 ^c^	0.97 ± 0.05 ^a^	1.14 ± 0.02 ^b^	1.14 ± 0.06 ^b^	1.00 ± 0.02 ^a^	
102.	*n*-Pentacosane	2500	2500	2500	6.13 ± 0.20 ^e^	4.80 ± 0.28 ^b^	5.63 ± 0.08 ^d^	5.11 ± 0.28 ^c^	4.18 ± 0.07 ^a^	alkane
103.	*n*-Hexacosane	2600	2600	2600	0.43 ± 0.01 ^d^	0.36 ± 0.02 ^b^	0.40 ± 0.01 ^c^	0.37 ± 0.02 ^b^	0.32 ± 0.01 ^a^	alkane
104.	Benzoic acid hexadecyl ester	2665	2664 ^F^		0.09 ± 0.00 ^a^	0.09 ± 0.01 ^a^	0.10 ± 0.00 ^a^	0.09 ± 0.02 ^a^	0.12 ± 0.01 ^b^	
105.	*n*-Heptacosane	2700	2700	2700	15.40 ± 0.40 ^c^	13.93 ± 0.79 ^b^	14.66 ± 0.17 ^bc^	13.90 ± 1.15 ^b^	11.21 ± 0.26 ^a^	alkane
106.	Benzyl oleate	2757	2758 ^G^		0.10 ± 0.02 ^b^	tr ^a^	0.05 ± 0.01 ^a^	0.14 ± 0.03 ^c^	0.16 ± 0.02 ^c^	
107.	Benzyl linoleate	2767	2764 ^E^		1.74 ± 0.02 ^a^	1.72 ± 0.08 ^ab^	1.85 ± 0.01 ^b^	2.09 ± 0.07 ^c^	2.17 ± 0.06 ^c^	
108.	Benzyl linolenate	2778	2775 ^E^		0.30 ± 0.00 ^a^	0.27 ± 0.01 ^a^	0.26 ± 0.04 ^a^	0.39 ± 0.01 ^b^	0.53 ± 0.02 ^c^	
109.	*n*-Octacosane	2800	2800	2800	0.14 ± 0.00 ^a^	0.39 ± 0.01 ^b^	0.41 ± 0.03 ^b^	0.59 ± 0.04 ^c^	0.54 ± 0.03 ^c^	alkane
110.	Squalene	2835	2836 ^E^	2865 ^E^	0.10 ± 0.00 ^a^	0.14 ± 0.03 ^b^	0.15 ± 0.00 ^b^	0.35 ± 0.02 ^c^	0.88 ± 0.02 ^d^	
111.	*n*-Nonacosane	2900	2900	2900	7.27 ± 0.11 ^c^	7.03 ± 0.31 ^c^	6.22 ± 0.08 ^b^	5.64 ± 0.20 ^a^	6.06 ± 0.18 ^b^	alkane
112.	1-Hexacosanol	2912	2906		0.08 ± 0.01 ^a^	0.16 ± 0.01 ^c^	0.14 ± 0.01 ^bc^	0.15 ± 0.02 ^bc^	0.15 ± 0.04 ^bc^	
113.	*n*-Triacontane	3000	3000	3000	0.17 ± 0.01 ^a^	0.32 ± 0.02 ^d^	0.24 ± 0.01 ^b^	0.23 ± 0.01 ^b^	0.27 ± 0.02 ^c^	alkane
114.	γ-Tocopherol	3075	3074			tr ^ab^	tr ^a^	0.05 ± 0.01 ^b^	0.12 ± 0.01 ^c^	
115.	*n*-Untriacontane	3100	3100	3100	6.73 ± 0.20 ^c^	6.46 ± 0.38 ^b^	4.41 ± 0.13 ^a^	4.51 ± 0.08 ^a^	6.22 ± 0.24 ^b^	alkane
116.	Vitamin E (D-α-tocopherol)	3154	3149 ^E^		0.14 ± 0.00 ^a^	0.50 ± 0.03 ^c^	0.25 ± 0.01 ^b^	0.99 ± 0.01 ^d^	1.76 ± 0.11 ^e^	
Total compounds identified/representing % of total volatiles					105/77.47	100/74.12	104/76.07	93/74.10	92/76.69	
Grouped compounds (%)										
	Aromatics				12.67	12.55	13.64	14.16	14.32	
	Aliphatic hydrocarbons (alkanes)				54.93	47.65	48.44	45.72	41.47	
	Aromatic hydrocarbons				0.04	0.04	0.04	0.18		
	Monoterpene hydrocarbons				0.18	0.25	0.21	0.16	0.15	
	Oxygenated monoterpenes				1.34	2.35	2.39	2.81	3.97	
	Oxygenated sesquiterpenes				0.31	0.45	0.44	0.43	0.41	
	Alcohols and esters				2.30	2.66	2.37	3.02	5.58	
	Aldehydes, ketones and lactones				3.14	4.44	4.95	3.37	2.75	
	Acids				1.27	1.71	1.75	1.44	3.12	
	Amides				0.58	0.70	0.82	0.86	1.32	
	Diterpenoids				0.05	0.04	0.05	0.06	0.23	
	Triterpenoids and tocopherols				0.24	0.68	0.42	1.39	2.76	
	Phenylpropanoids and phenols				0.12	0.16	0.14	0.14	0.40	
	Other (oxanes, heteroaromatics, etc.)				0.35	0.48	0.46	0.42	0.44	

^#^ Compounds are listed in order of their elution from nonpolar BPX-5 capillary column. ^A^ Identified on the basis of GC-TOF/MS spectra based on comparison with NIST, MainLib, RepLib and Adams libraries and calculated KI with those reported in Adams and Nist, PubChem and ChemSpider databases. ^B^ Kováts retention indices calculated against C_7_-C_30_ *n*-alkanes on BPX-5 column. ^C^ Kováts retention indices on nonpolar DB-5 column reported in literature [27]. ^D^ Kováts retention indices calculated on polar carbowax 20 M column reported in literature (Flavornet, http://flavornet.org/flavornet.html) (accessed on 19 July 2021). ^E^ Kováts retention indices from the database http://webbook.nist.go (accessed on 19 July 2021). ^F^ Kováts retention indices from the database https://pubchem.ncbi.nlm.nih.gov (accessed on 19 July 2021). ^G^ Kováts retention indices from the database http://www.chemspider.com (accessed on 19 July 2021). ^1^ Odour description from http://flavornet.org. (accessed on 23 October 2021) (and without number indications) [25]; ^2^ Odour description from SAFC^®^ Flavors & Fragrances (www.safcglobal.com) (accessed on 23 October 2021) [26]; tr—trace (≤ 0.04%); RSD%, average coefficient of variance of individual compounds. * *n*-Decane corresponds the percentage content of an alkane extracted initially from the plant material. Values within rows followed by the same superscript letter (a–e) do not differ statistically at *p* < 0.05 (Duncan test).

## Data Availability

Data available from the authors upon request.
